# Polydopamine‐Mediated Grafting of Cationic Polymer Brushes for Adsorption of Fluorinated Compounds

**DOI:** 10.1002/chem.202503580

**Published:** 2026-01-19

**Authors:** Agnes C. Morrissey, Federica Sbordone, Fred Pashley‐Johnson, Aaron S. Micallef, Bart van de Worp, Neomy Zaquen, Prasanna Egodawatta, Laura Delafresnaye, Lukas Michalek, Christopher Barner‐Kowollik

**Affiliations:** ^1^ School of Chemistry and Physics Centre for Materials Science Queensland University of Technology (QUT) Brisbane Queensland Australia; ^2^ Polymer Chemistry Research Group Faculty of Science Ghent University Ghent Belgium; ^3^ Central Analytical Research Facility Queensland University of Technology (QUT) Brisbane Queensland Australia; ^4^ Lapinus ROCKWOOL B.V. Roermond The Netherlands; ^5^ School of Civil and Environmental Engineering Queensland University of Technology (QUT) Brisbane Queensland Australia; ^6^ Department of Chemical Engineering Stanford University Stanford California USA; ^7^ Institute of Functional Interfaces (IFG) Karlsruhe Institute of Technology (KIT) Eggenstein‐Leopoldshafen Germany

**Keywords:** adsorption, dopamine, functional surface, quaternary amine

## Abstract

Understanding the adsorption behavior of charged molecular species at functionalized polymer interfaces is critical for advancing surface science and material design. Building upon recent advances in polydopamine (PDA)‐mediated polymer grafting, we investigated electrostatic adsorption at cationic polymer brush surfaces. Using a grafting‐to approach, we covalently attached poly(2‐trimethylammonioethyl methacrylate chloride) (PTMAEMA) to PDA‐coated stonewool fibers and characterized the resulting charged interface via x‐ray photoelectron spectroscopy (XPS), scanning electron microscopy (SEM), and thermogravimetric analysis (TGA). We employed perfluorooctanoic acid (PFOA) as a model anionic adsorbate to investigate binding at a quaternary amine‐functionalized surface. Batch equilibrium sorption studies revealed concentration‐dependent adsorption kinetics, achieving three times higher binding affinity for PTMAEMA‐functionalized fibers at concentrations ranging from 0.5 to 5.0 g L^−^^1^, with equilibrium reached within 10 min. XPS analysis confirmed the successful surface functionalization with distinct nitrogen environments at 395.5 eV (PDA) and 398.5 eV (quaternary nitrogen), while thermogravimetric data indicated an organic loading of close to 28%. Our findings demonstrate that PDA‐mediated polymer grafting provides a versatile platform for creating well‐defined charged interfaces with tunable adsorption characteristics, and provides a methodology to explore fundamental adsorption phenomena at polymer‐liquid interfaces.

## Graphical abstract

We introduce a versatile approach to fabricate tunable charged interfaces by functionalizing stonewool fibers with a polydopamine (PDA)–quaternary amine coating via a grafting‐to method. The modified surfaces exhibit rapid, concentration‐dependent adsorption of anionic species driven by electrostatic interactions.

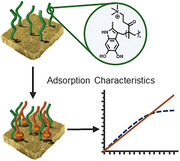

## 1 Introduction

Functionalized polymer surfaces provide powerful platforms for investigating fundamental interfacial phenomena such as molecular adsorption, charge transfer, and electrostatic binding [1]. The ability to precisely control surface chemistry through polymer modification enables systematic studies on how molecular architecture and charge density influence adsorption thermodynamics and kinetics [2]. Recent advances in surface‐initiated polymerization and grafting techniques have expanded the toolkit for creating well‐defined polymer interfaces [3]. Yet, the relationship between the surface charge distribution and molecular adsorption behavior remains not fully understood, particularly for highly charged polymer brush systems.

Traditional methods for preparing polymer‐functionalized surfaces, including self‐assembled monolayers (SAMs) [4], layer‐by‐layer (LbL) assembly [5], and plasma treatment [6], often require specific substrate chemistries or multistep surface preparation protocols. SAMs, while providing excellent molecular‐level control, are typically limited to noble metal or oxide surfaces and involve complex synthetic procedures for functional thiols or silanes. LbL assembly offers versatility, but results in physically adsorbed multilayers that may lack long‐term stability under harsh conditions. Plasma treatments can modify virtually any surface but offer limited control over the chemical functionality introduced and may cause surface degradation. In contrast, polydopamine (PDA) has emerged as a remarkably versatile platform for universal surface modification due to its ability to adhere to virtually any substrate under mild aqueous conditions [7]. Upon oxidation, dopamine undergoes self‐polymerization to form a thin uniform coating containing catechol, amine, and imine functional groups, which can serve as reactive handles for subsequent functionalization [8]. While the adhesion mechanism and polymerization pathway of PDA are established [7], the precise molecular structure of PDA remains a subject of ongoing investigation, with evidence supporting both covalent polymeric and supramolecular aggregate models [9, 10]. Nevertheless, the practical utility of PDA coatings for surface functionalization is firmly demonstrated across numerous applications [11, 12, 13].

In 2022, Stenzel and colleagues demonstrated that stable free radical species within PDA—specifically carbon‐centered radicals (CCRs) and semiquinone radicals (SQRs)—can serve as termination sites for propagating vinyl polymer chains, enabling a grafting‐to approach for polymer attachment [14]. Their methodology leverages the universal adhesion properties of PDA while providing covalent polymer grafting through radical–radical coupling. The study established the underpinning mechanism via electron paramagnetic resonance (EPR) spectroscopy and demonstrated the successful grafting of various vinyl polymers onto PDA‐coated surfaces. Despite these advances, several fundamental questions remain open. The relationship between grafting density, polymer brush conformation, and functional performance has not been systematically explored for PDA‐grafted systems. Moreover, while the grafting‐to mechanism is established, the subsequent adsorption behavior and interfacial properties of these PDA‐polymer hybrid materials have not been characterized.

Our research group has previously explored PDA‐coated surfaces for covalent molecular attachment and for metal ion adsorption from stormwater [15, 16]. However, the adsorption behavior of charged organic species via cationic polymer brush interfaces created via PDA‐mediated grafting has not been investigated. The present study addresses this gap by combining the grafting‐to methodology with systematic adsorption studies, thereby extending the utility of PDA‐polymer hybrid materials to a new class of adsorbates. Herein, we advance the PDA‐mediated radical grafting approach to investigate the fundamental adsorption behaviors of charged polymer brush surfaces. We selected poly(2‐trimethylammonioethyl methacrylate chloride) (PTMAEMA) as a model cationic polymer due to its intrinsic positive charge provided by the quaternary ammonium functionality. Using stonewool—a porous fibrous mineral material—as a three‐dimensional substrate, we fabricated PDA‐coated surfaces and subsequently grafted PTMAEMA via the above‐mentioned free‐radical termination mechanism. Our system allowed us to study the physical chemistry of electrostatic adsorption at a well‐defined charged interface.

**SCHEME 1:**
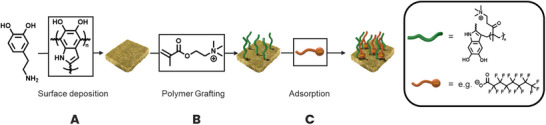
Schematic overview of the surface attachment of PDA onto stonewool fibers and the functionalization of the surface via a grafting‐to approach for the adsorption of anionic molecules. (A) Polymerization of dopamine to form a PDA coating; (B) free‐radical polymerization of TMAEMA in solution that is subsequently grafted to the PDA through termination; (C) adsorption of anionic molecules via electrostatic interactions.

To investigate the adsorption characteristics of our functionalized surfaces, we employed perfluorooctanoic acid (PFOA) as a model anionic adsorbate. PFOA is particularly well‐suited for our study due to its strong anionic character (pKa close to 2.8) [17], high solubility across a range of pH values, and straightforward quantification via ^1^ ^9^F NMR spectroscopy [18]. By varying the adsorbate concentration and monitoring the adsorption kinetics, we can extract the fundamental parameters that describe electrostatic binding at the quaternary amine‐functionalized interface. The surface functionalization pathway and adsorption study is highlighted in Scheme 1.

Our study addresses several fundamental questions: (i) How does covalent grafting of charged polymer brushes via PDA mediation affect surface properties and adsorption capacity compared to unmodified substrates? (ii) What are the kinetics and equilibrium behavior of electrostatic adsorption at these interfaces? (iii) How does adsorbate concentration influence binding efficiency and capacity? Through comprehensive surface characterization (XPS, SEM, TGA) combined with systematic adsorption measurements, we demonstrate that PDA‐mediated polymer grafting provides a versatile route for creating charged interfaces with well‐defined adsorption properties. Our findings establish a framework for understanding (electrostatic) adsorption at polymer brush surfaces and highlights the potential of the PDA radical coupling methodology for creating functional interfaces for physical chemistry studies.

## 2 Results and Discussion

Upon exposure to mildly oxidizing conditions or alkaline pH, the catechol of dopamine is readily transformed into a quinone functionality that can then undergo polymerization to form PDA (Scheme 1A) [19], a nonperiodic polymeric structure comprised of many monomer and linkage types [20]. In 2022, Nothling et al. demonstrated that stable radicals within the PDA structure are able to terminate free radical polymerization processes, thus establishing a covalently bound polymer brush on the surface [14]. In an adaptation of this protocol, we initially polymerized dopamine onto silicon wafers in a Tris buffer. Subsequently, 2‐trimethylammonioethyl methacrylate chloride (TMAEMA) was thermally polymerized in an aqueous solution in the presence of the coated wafers, grafting the polymer to the wafer surface through the aforementioned termination mechanism (Scheme 1B). The formed polymer brushes refer to a grafted polymer surface rather than a specific brush density [21]. In the referenced study, a PDA thickness of 10.9 ± 2.6 nm on glass slides is reported, along with an increase of 24.3 ± 1.5 nm after deposition of the monomer (N‐isopropylacrylamide). We assume a similar film thickness for the film produced when altering the monomer and therefore continued investigating the chemical composition of the fabricated film via x‐ray photoelectron spectroscopy (XPS) [14]. The recorded XPS spectra of the coated wafers (Figures S1–S4) revealed that the grafting procedure was successful, as indicated by the presence of two peaks in the N 1s high‐resolution spectra at 396.2 eV, which can be attributed to the nitrogen in the PDA coating, and 399.8 eV, which is associated with the quaternary nitrogen in the polymer strands of the coated samples.

**FIGURE 1:**
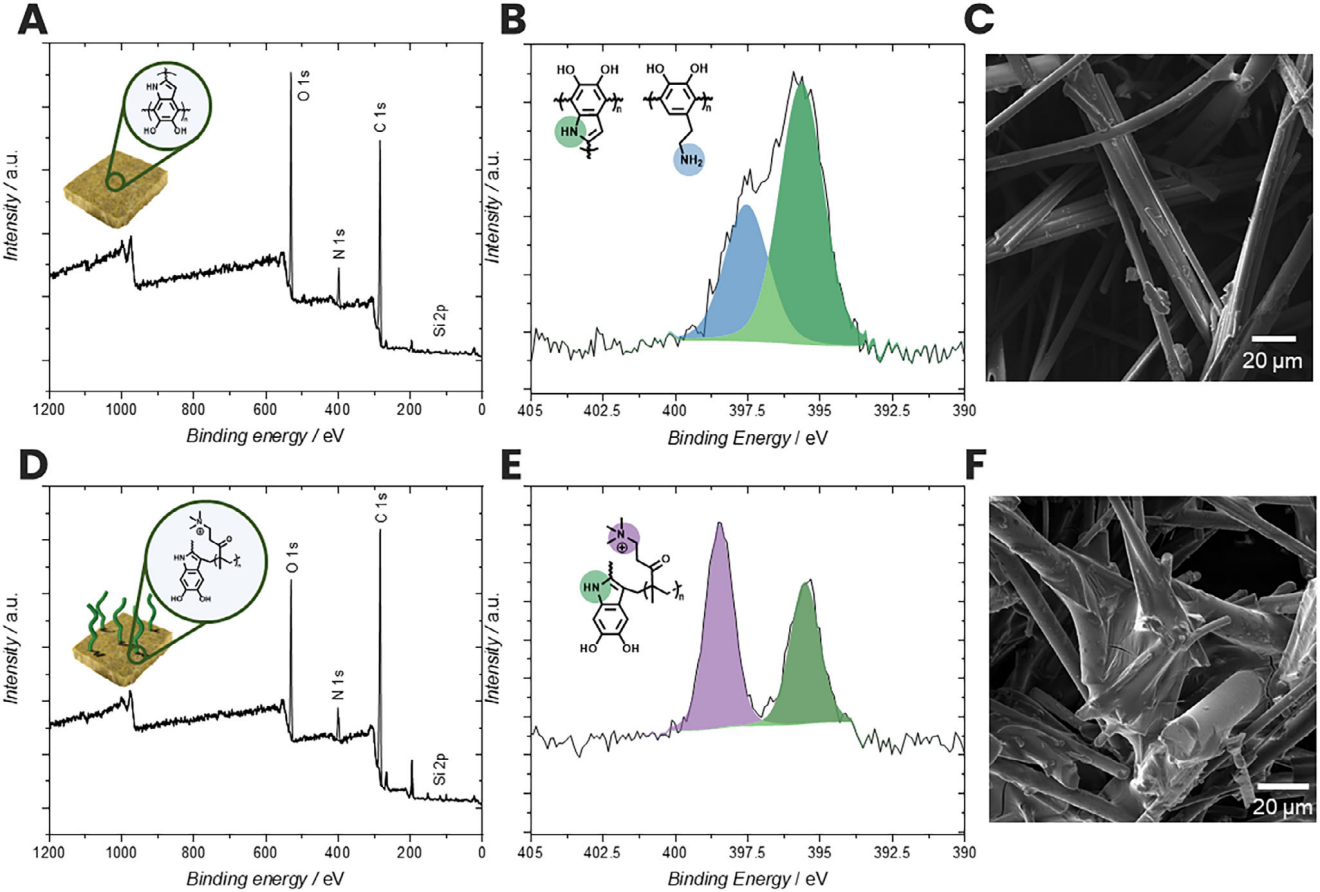
(A) Wide‐scan XPS spectra of the PDA‐coated stonewool fibers and (B) corresponding N 1s high‐resolution spectrum, as well as the SEM micrograph of the coated fiber surface (C). (D) Wide‐scan spectra of the PDA‐coated stonewool fibers after polymer grafting and (E) corresponding N 1s high‐resolution spectrum as well as the corresponding SEM micrograph of the polymer‐grafted surface (F).

Encouraged by these findings, we applied a similar coating protocol to the stonewool fibers (the experimental procedure is detailed in section 3 of the Supporting Information). The wide‐scan XPS spectra of the PDA‐coated fibers and the functionalized fibers, as well as their corresponding N 1s high‐resolution spectra, are shown in Figure 1A,B (PDA‐coated surface) and Figure 1D,E (polymer‐grafted surface). In Figure 1B, two main components of the N 1s peak can be observed. These peaks are associated with an aliphatic primary amine (397.5 eV, 34.39%, blue) and a conjugated secondary amine (395.6 eV, 65.61%, green). Upon addition and polymerization of the quaternized amine‐containing monomer, the chemical environment changes, which is apparent in the N 1s high‐resolution spectrum of the polymer grafted surface (Figure 1E). Two distinct peaks can be observed, corresponding to the nitrogen contained in the PDA at 395.5 eV (42.97%, green) and the quaternary nitrogen contained in the polymer strands at 398.5 eV (57.03%, purple).

The successful coating of the fibers is corroborated by scanning electron microscopy (SEM, Figure 1C, F). The PDA‐coated samples display minimal morphological changes compared to the pristine fibers (Figure S5). In contrast, upon polymerization of TMAEMA, large organic structures that were susceptible to beam damage were observed uniformly across the fiber sample, indicating the successful functionalization of the PDA coating. In addition, thermogravimetric analysis (TGA) of the coated and pristine fibers (Figure S6) reveals a mass loss of approximately 28% for the polymer‐coated structures, in contrast to less than 3% for the pristine and PDA‐coated fibers, thus indicating that a significant amount of organic material has been successfully attached to the surface. Additionally, the TGA studies revealed that the coatings are stable up to temperatures of 200°C. Furthermore, we utilized BET measurements to determine the surface area of the differently functionalized fibers. The surface areas were close to 1.7579 m^2^ g^−1^ for the pristine fibers, 7.0914 m^2^ g^−1^ for the PDA‐coated fibers, and 5.3781 m^2^ g^−1^ for the polymer grafted fibers. Observing an increased surface area upon deposition of the PDA coating is expected due to the roughness of the coating [22]. These results further support the successful surface coating and reveal that the surface area decreases upon polymer grafting of TMAEMA.

After confirming the successful coating of the stonewool fibers, we investigated the ability of the functionalized fibers to adsorb negatively charged fluorine‐containing monomers. Here, we selected PFOA as a model compound, as changes in F concentration can be tracked straightforwardly via ^19^F NMR analyses, making the investigation of the amount adsorbed readily accessible. A detailed description of the ^19^F NMR studies is given in Section 9 of the Supporting Information as well as exemplary ^19^F NMR spectra in Figure S7 and the calibration curve used to calculate concentrations in Figure S8 [18]. Initially, we investigated the adsorption equilibrium of a 2.5 g L^−1^ PFOA solution when treated with the polymer grafted stonewool fibers or pristine stonewool fibers, revealing that equilibrium was reached after 10 min, and consequently, further experimental data were recorded up to 3 h (Figure S9). We subsequently studied the kinetic behavior of PFOA adsorption through batch equilibrium sorption experiments at three different concentrations of PFOA in water (0.5, 2.5, and 5 g L^−1^). Each concentration was tested in triplicate to assess experimental variability. The adsorption of PFOA was evaluated via ^19^F nuclear magnetic resonance (NMR) spectroscopy.

**FIGURE 2:**
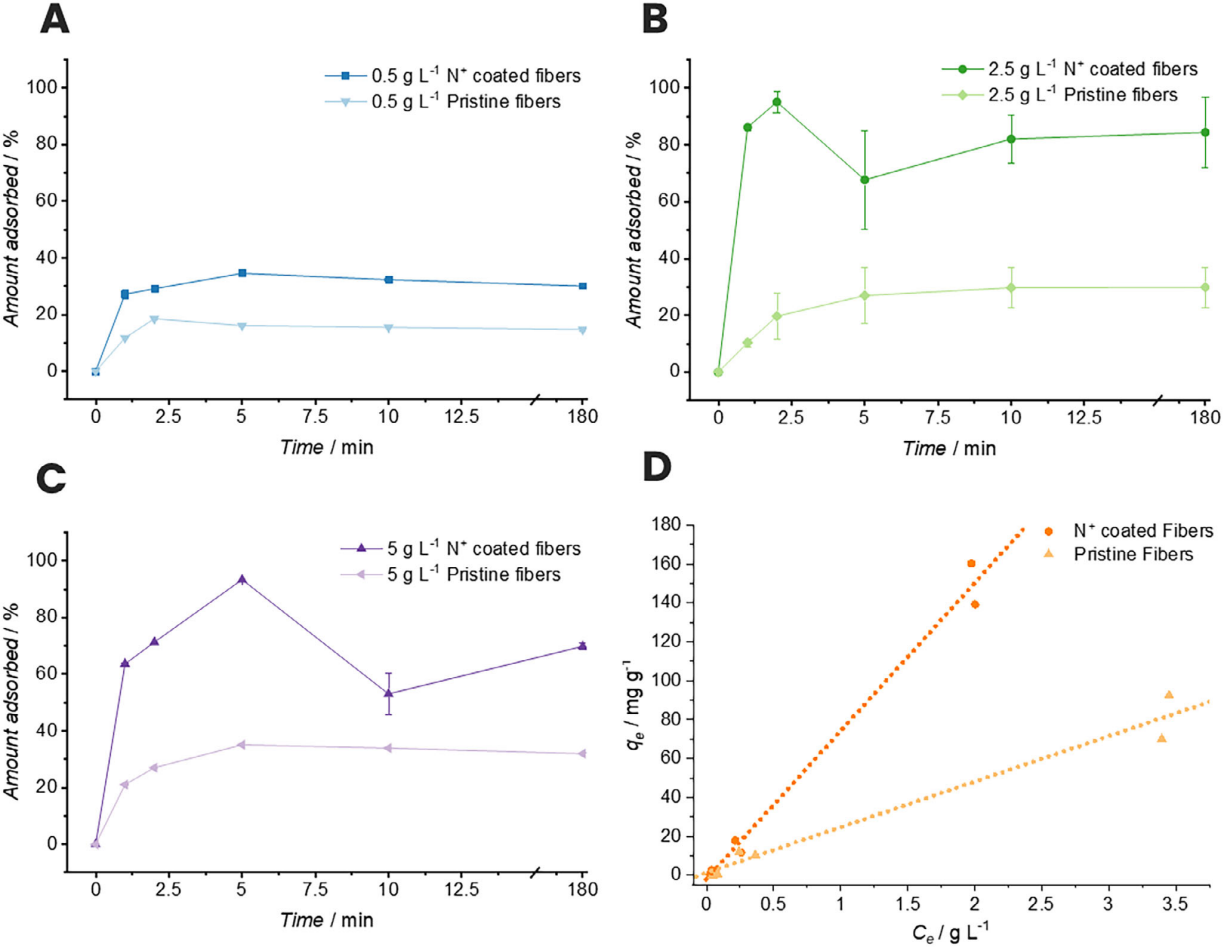
Time‐dependent adsorption of PFOA at three different concentrations of the polymer grafted functionalized fibers versus the pristine fibers (A — 0.5, B — 2.5, and C — 5 g L^−1^). The datapoints are the mean value of triplicates and the error bars represent the standard error associated with the triplicate experiments. The adsorption isotherm of the polymer grafted fibers versus the pristine fibers fitted to the Langmuir model is shown in viewgraph (D).

Figure 2 shows the results of PFOA adsorption by the coated fiber surface at three concentrations. As expected, the lowest concentration (Figure 2A, 0.5 g L^−1^, blue) shows the lowest percentage of PFOA adsorbed by the polymer‐grafted and pristine fibers. Since the concentration equilibrium between the PFOA molecules and the adsorbent (stonewool fibers) is lower, there is less driving force and fewer PFOA molecules to be adsorbed. The amount adsorbed by the polymer grafted fibers reaches equilibrium at 5 min, with an adsorption of 35% of the initial PFOA concentration, in comparison to the pristine fibers, which reach a maximum adsorption of 19%. At higher concentrations of PFOA, the equilibrium adsorption achieved is as high as 93% (Figure 2C, 5 g L^−1^, purple) and 95% (Figure 2B, 2.5 g L^−1^, green), indicating that a high adsorption capacity can be reached. For these higher concentrations, we again observed a higher adsorbed amount for the coated fibers in comparison to the pristine fibers (5 g L^−1^ — 35%, 2.5 g L^−1^ — 30%). In addition, to gain a deeper understanding of the adsorption behavior of PFOA, we conducted a concentration‐dependent study utilizing a 0.05, 0.1, 2.5, 5, 6.5, and 8 g L^−1^ concentrated aqueous solution and determined the amount of PFOA adsorbed on the polymer grafted stonewool adsorbent after 10 min. The Langmuir plots of the pristine and polymer grafted fibers are featured in Figure 2D. These results reveal that the adsorption at the employed concentrations is in the linear region and the adsorption maximum has not yet been reached for the pristine and the polymer grafted fibers. Quantitative analysis of the linear regime of the Langmuir isotherms shows that the polymer‐grafted fibers exhibit a three times steeper slope compared to the pristine fibers (76.1 mg g^−^^1^ (g L^−^^1^)^−^^1^ for N^+^ coated fibers and 23.5 mg g^−^^1^ (g L^−^^1^)^−^^1^ for pristine fibers), demonstrating a superior adsorption capacity at all tested concentrations.

The enhanced adsorption capacity correlates with the increase in surface area following PDA coating and polymer grafting. The previously mentioned BET analysis revealed that polymer‐grafted fibers possess a surface area of approx. 5.3781 m^2^ g^−^^1^ compared to 1.7579 m^2^ g^−^^1^ for pristine fibers, which is a three‐fold increase that parallels the three‐fold enhancement in adsorption slope. While this correlation suggests surface area contributes to the improved performance, literature precedent indicates that quaternary ammonium functionalization can provide adsorption enhancements of 10–15 times beyond surface area effects alone through specific electrostatic interactions between cationic sites and anionic head groups [23]. The rapid equilibration kinetics (within 10 min) observed in our system are consistent with electrostatic binding mechanisms reported for other quaternary ammonium‐functionalized adsorbents. Although the current data cannot decouple surface area contributions from specific electrostatic effects, the grafting methodology provides a practical route to enhanced adsorption capacity. Future investigations on ionic strength variation or pH‐dependent measurements will further elucidate the relative contributions of surface area and electrostatic binding. Additionally, the multi‐anion systems should be further studied to determine the selectivity coefficients for different anions present, especially regarding practical applications such as common anions present in polluted water matrices.

## 3 Conclusion

We introduce a facile surface modification technique that enables the grafting of poly‐cationic polymer chains to a PDA‐coated surface, resulting in a three‐fold enhancement in both surface area and adsorption capacity for PFOA as a model charged compound. We demonstrate that both silicon wafers and stonewool fibers (a fibrous mineral‐based material) can be readily coated. Subsequently, we evaluated the ability of the coated materials to adsorb PFOA through concentration‐dependent adsorption studies. These results revealed that our polymer‐grafted fibers exhibit rapid equilibration and significantly improved adsorption behavior compared to the uncoated stonewool fibers. Our study establishes a straightforward method for fabricating grafted polymer brushes onto a stonewool surface to create charged functional interfaces with tunable adsorption characteristics. While the introduced fabrication method is useful for industrial applications, as it utilizes a simple, inexpensive, and scalable approach, further enhancement of the functionalized surface should be targeted at the regeneration and reusability of adsorbent materials. Recent literature demonstrates several strategies for desorbing PFAS from quaternary ammonium‐functionalized materials, including treatment with concentrated salt solutions (e.g., NaCl or NH_4_Cl), solvent washing with methanol or ethanol mixtures, or electrochemical regeneration methods [24, 25]. The covalent attachment of polymer brushes through PDA‐mediated radical coupling is expected to provide greater stability toward regeneration cycles compared to physically adsorbed coatings.

## Conflicts of Interest

The authors declare no conflicts of interest.

## Supporting information




**Supplementary File 1**: chem70701‐sup‐0001‐SuppMat.pdf.
