# Supplementary material for: Polydopamine‐Mediated Grafting of Cationic Polymer Brushes for Adsorption of Fluorinated Compounds
Source: Chemistry. 2026 Jan 19;32(12):e03580. doi: 10.1002/chem.202503580 (PMC13037358; doi:10.1002/chem.202503580)
Supplement: Supplementary file 1 — chem70701‐sup‐0001‐SuppMat.pdf. [file CHEM-32-e03580-s001.pdf]

Supporting Information

Polydopamine-Mediated Grafting of Cationic Polymer Brushes for Adsorption of Fluorinated Compounds

Agnes C. Morrissey,[#] Federica Sbordone,[#] Fred Pashley-Johnson, Aaron S. Micallef, Bart van de Worp, Neomy Zaquen, Prasanna Egodawatta, Laura Delafresnaye, Lukas Michalek,* Christopher Barner-Kowollik*

A. C. Morrissey, Dr. F. Sbordone, Dr. F. Pashley-Johnson, Dr. L. Michalek, Dr. L. Delafresnaye, Prof. Dr. C. Barner-Kowollik
School of Chemistry and Physics
Queensland University of Technology (QUT)
2 George Street, 4000 Brisbane, Queensland, Australia
E-mail: christopher.barnerkowollik@qut.edu.au

Dr. F. Pashley-Johnson
Department of Organic and Macromolecular Chemistry, Polymer Chemistry Research Group, Centre of Macromolecular Chemistry (CMaC), Faculty of Science
Ghent University
Krijgslaan 281 (S4-Bis), 9000 Ghent, Belgium

A/Prof. A. S. Micallef
Central Analytical Research Facility
Queensland University of Technology (QUT)
2 George Street, 4000 Brisbane, Queensland, Australia

D. Ghijzen, B. van de Worp, Dr. N. Zaquen
Lapinus, ROCKWOOL B.V.
Delfstoffenweg 2, 6045JH Roermond, The Netherlands

A/Prof P. Egodawatta
School of Civil and Environmental Engineering
Queensland University of Technology (QUT)
2 George Street, 4000 Brisbane, Queensland, Australia

Dr. L. Michalek
Department of Chemical Engineering
Stanford University
Stanford, CA, USA

Prof. Dr. C. Barner-Kowollik
Institute of Functional Interfaces (IFG)
Karlsruhe Institute of Technology (KIT)
Hermann-von-Helmholtz-Platz 1, 76344 Eggenstein-Leopoldshafen, Germany

[#]These two authors have contributed equally.

Contents

1 **Materials** 2
2 **Instrumentation** 2
3 **Surface Coating** 3
4 **X-Ray Photoelectron Spectroscopy (XPS)** 4
5 **Scanning Electron Microscopy** 9
6 **Thermogravimetric Analysis (TGA)** 10
7 **Brunauer–Emmett–Teller (BET) measurements** 11
8 **Adsorption Experiments** 12
9 **Nuclear Magnetic Resonance (NMR) Spectroscopy** 12
10 **Adsorption Isotherm** 15
11 **References** 16

1 Materials

All chemicals and solvents were used as received from the supplier without further purification.

Methanol (Thermo Fisher), ethanol (Thermo Fisher), water (Milli-Q, Merck), tris(hydroxymethyl)aminomethane (ACS reagent grade, Merck), dopamine hydrochloride (98%, Sigma-Aldrich), [2-(methacryloyloxy)ethyl]trimethylammonium chloride solution (75 wt. % in H_2O, Sigma-Aldrich), perfluorooctanoic acid (95%, Merck), 3,5-difluorobenzoic acid (97%, Sigma-Aldrich), chromium(III) acetylacetonate (97%, Sigma-Aldrich), 4,4'-Azobis(4-cyanopentanoic acid) (ABCPA) (98%, Thermo Fisher).

Deuterium oxide-d_2 (D_2O-d_2, 99.9%, Sigma-Aldrich) was utilized as a locking solvent for NMR measurements.

2 Instrumentation

Nuclear Magnetic Resonance (NMR) Spectrometry: ^{19}F spectra were recorded on a 400 MHz Bruker Neo spectrometer, equipped with a TBO-Probe (5 mm) with a dedicated ^{19}F channel and z-gradient (^{19}F 376.51 MHz) at 298 K.

X-ray photoelectron spectroscopy (XPS): XPS spectra were collected using a Kratos Axis Supra system operating with a monochromatic Al Kα source (1486.7 eV). Survey spectra and

high-resolution core-level spectra were collected with pass energies of 160 and 20 eV respectively and a step size of 0.1 eV. All XPS data were processed with CasaXPS. All spectra were calibrated by setting the C 1s peak to 285.00 eV.

Thermogravimetric Analysis (TGA): TGA was performed on a STA 449 F3 Jupiter from Netzsch. Samples were analyzed in aluminum oxide pans at a heating rate of 20 °C·min^{-1} from 20 °C to 800 °C under nitrogen atmosphere. The data was analyzed using TA Instruments Universal Analysis 2000 software (version 4.2E).

Brunauer-Emmet-Teller method (BET): Before the analysis samples were degassed under vacuum at 150 °C for 24 hours using a Micromecritics Smart VacPrep sample preparation system. After the degassing, the nitrogen adsorption and desorption isotherms were collected at the temperature of -196 °C on a Micromeritics 3Flex analyser. The single point total pore volume was calculated from the nitrogen amount adsorbed at a maximum relative pressure (p/p°) of 0.99 from the adsorption/desorption branch. The specific surface area was calculated using the Brunauer–Emmett–Teller (BET) method.

Scanning electron microscopy (SEM): SEM images were captured using a Tescan MIRA3 scanning electron microscope operating at 5 kV with a beam intensity of 8.0. Samples were coated with 4 nm platinum prior to imaging.

3 Surface Coating

Si wafers - polydopamine coating: Following slightly modified literature procedures,[1-2] 60 µL of 0.2 M dopamine solution in ethanol and 60 µL of 0.3 M Tris buffer solution were consecutively drop-cast onto a 1 x 1 cm Si wafer and left to react overnight at room temperature. The Si wafers were then thoroughly washed with Milli-Q water and ethanol.

Stonewool fibers – polydopamine coating: 20 cubes weighing 60 mg each of Stonewool fibers were immersed in 50 mL of a 1:1 (v/v) solution of 0.2 M dopamine and 0.3 M Tris buffer and left to react overnight at room temperature. The cubes were then thoroughly washed with Milli-Q water and ethanol.

Polymer grafting procedure: For the polymer grafting to dopamine coated Si wafers, the polymerization mixture was prepared in a 20:1 molar ratio of a 75% v/v [2-(methacryloyloxy)ethyl]trimethylammonium chloride solution in water (TMAEMA, 120.77 µmol, 25 µL) to initiator 4,4'-azobis(4-cyanopentanoic acid (ACPA 6.04 µmol, 1.69 mg) in 1 mL of Milli-Q water. For the grafting to dopamine coated Stonewool fibers, the polymerization

mixture was prepared in a 20:1 molar ratio of TMAEMA (483.09 μmol, 125 μL) to initiator (ACPA, 24.15 μmol, 6.76 mg) in 2.5 mL of Milli-Q water. The polydopamine coated surfaces (Si wafers and Stonewool cubes) were placed in a crimp vial and submerged in the monomer solution. After degassing by sparging the solution with argon for 5 min, the polymerization was carried out at 70 °C for 5 h. Subsequently, the Si wafers were rinsed with Milli-Q water and ethanol and dried under nitrogen flow and the Stonewool cubes were rinsed with Milli-Q water followed by ethanol and dried in a fan-assisted oven at 60 °C overnight.

4 X-Ray Photoelectron Spectroscopy (XPS)

We investigated the successful coating of the fibers with polydopamine and the subsequent polymer grafting with a [2-(methacryloyloxy)ethyl]trimethylammonium chloride solution via XPS. First, we studied both coating steps on Si wafers before coating the Stonewool fibers. The data (**Figure S1**) reveals the successful coating of the Si wafers with dopamine and **Figure S2-3** show the subsequent polymer grafted surface. In addition, the high-resolution spectrum of N1s shows bimodal character which can be assigned to the disparate environments of the nitrogen contained in dopamine and the quaternized amine (**Figure S3**). **Figure S4** shows the XPS wide-scan spectrum for comparison.

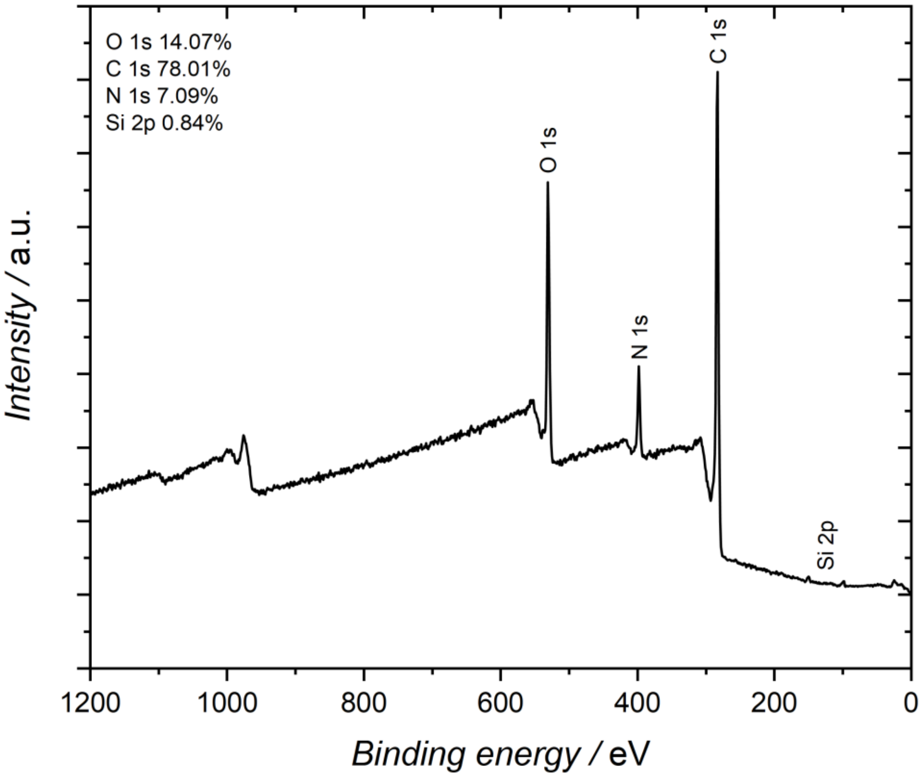

Figure S1. XPS wide-scan spectrum of a polydopamine (0.2 M) coated Si wafer.

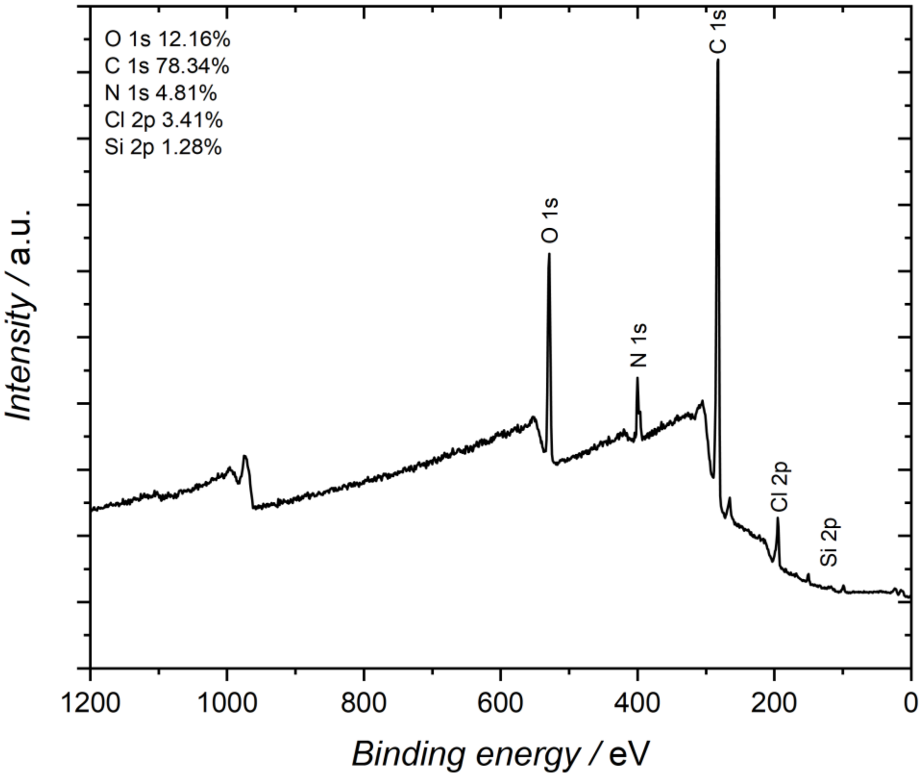

Figure S2. XPS wide-scan spectrum of a dopamine (0.2 M) coated Si wafer after a grafting to functionalization with TMAEMA (0.1 M).

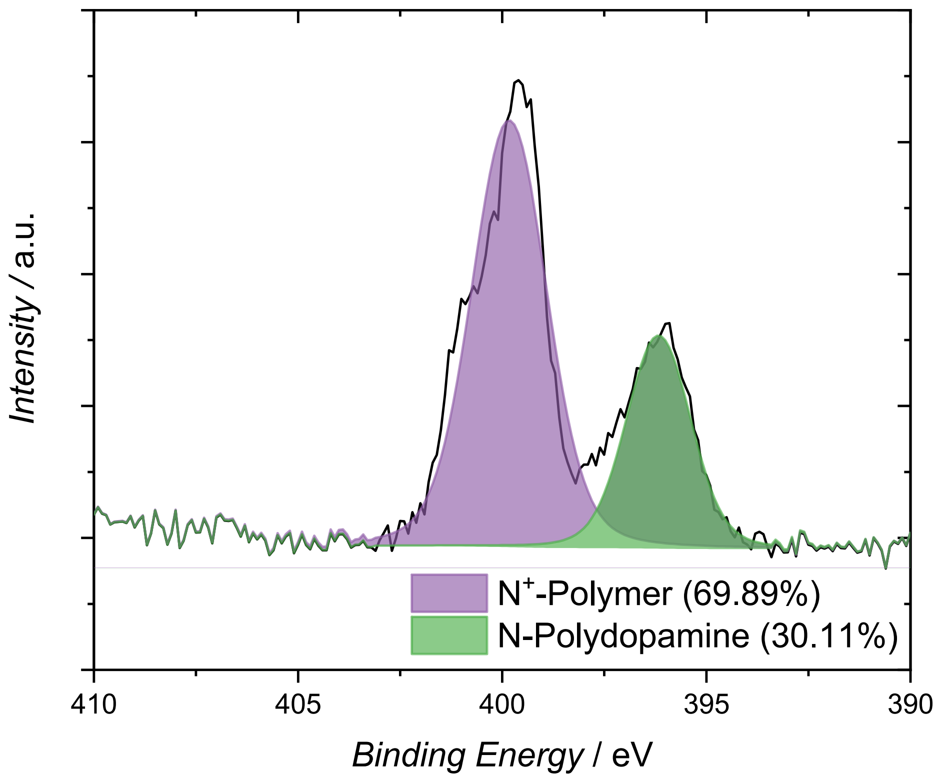

Figure S3. XPS high resolution spectrum of N 1s of the polymer grafted surface (Si wafer). N 1s reveals two components, i.e. the conjugated secondary amine in the dopamine backbone at 396.2 eV and the quaternary amine at 399.8 eV. The ratio between the dopamine N and the quaternary N is 1:0.43.

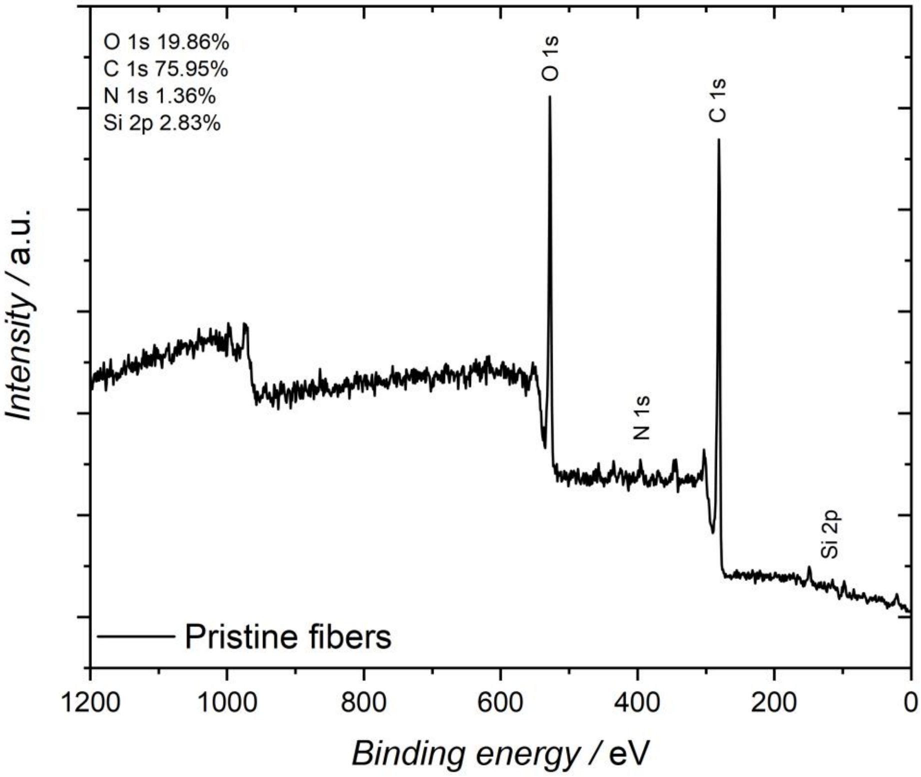

Figure S4. XPS wide-scan spectrum of pristine fibers.

5 Scanning Electron Microscopy

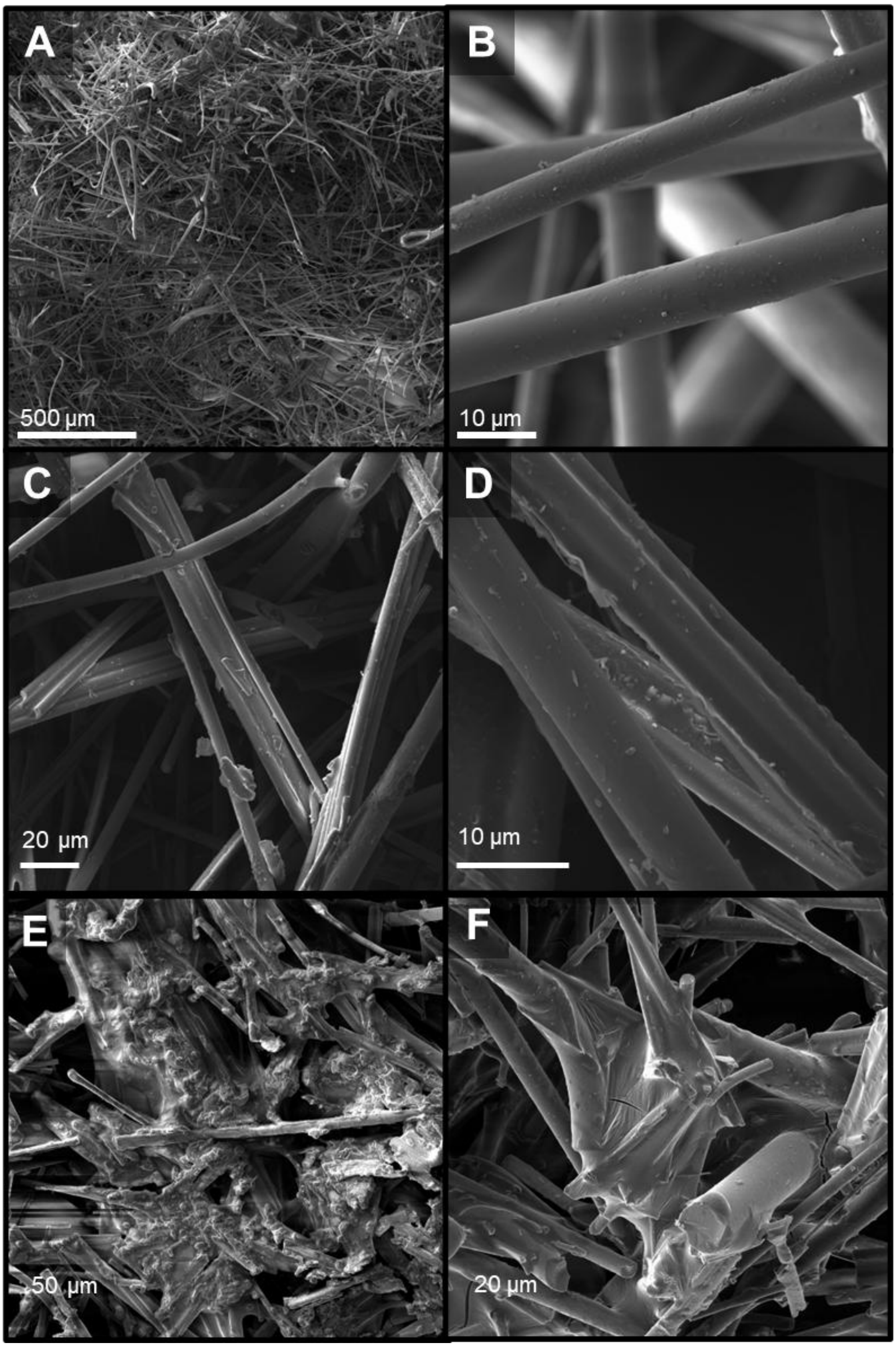

Figure S5. SEM micrographs of (A, B) pristine stonewool fibers; (C, D) stonewool fibers coated with dopamine; and (E, F) polymer grafted stonewool fibers. All samples were coated with 4 nm Pt before imaging.

6 Thermogravimetric Analysis (TGA)

To investigate the successful coating of the fibers TGA measurements were conducted. The TGA traces are shown in **Figure S6**. The TGA investigations revealed that there is more mass loss on the polymer grafted sample, specifically 28.13%, which is more than for the pristine fibers which exhibited a mass loss of 2.81% as expected. **Table S1** summarizes the mass loss after 20°C to 800°C temperature ramp.

Table S1. Mass remaining of the pristine and coated fibers after a temperature ramp of 20°C to 800°C over 40 minutes.

Fiber	Mass remaining [%] at 800°C
Pristine	97.19
Polydopamine	99.32
Polymer grafted	71.87

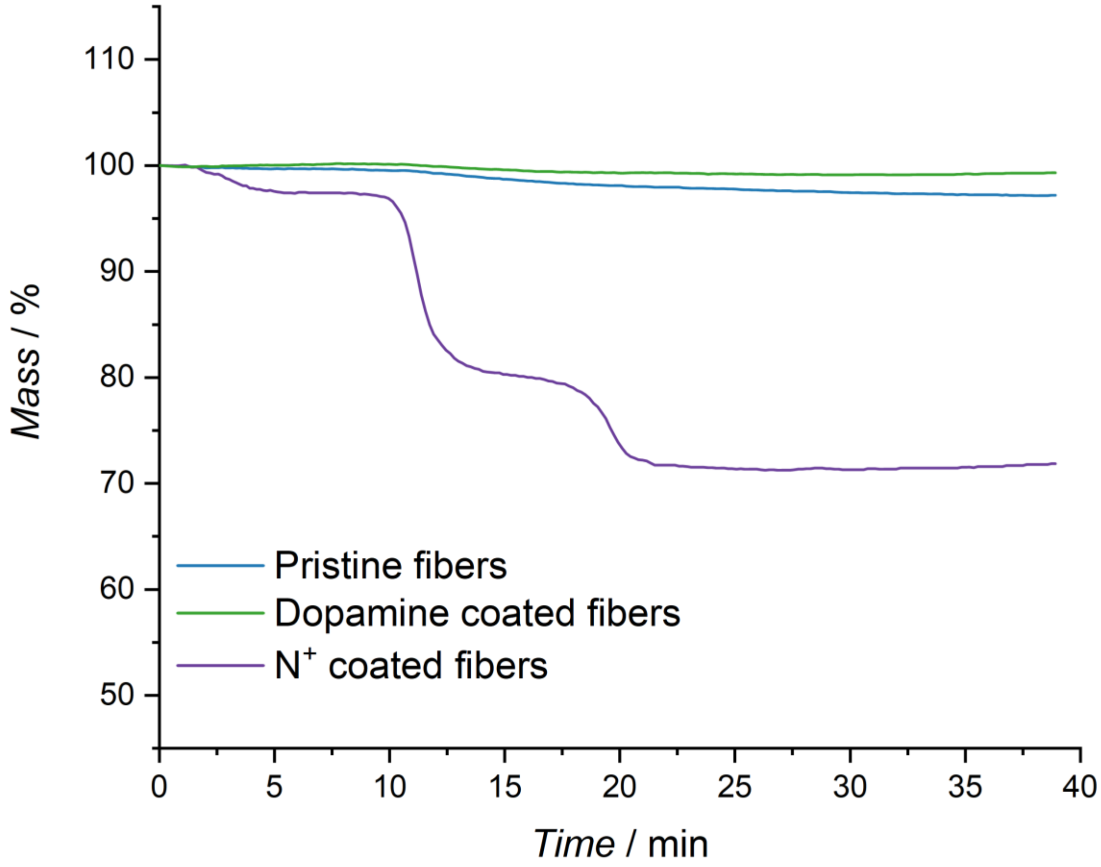

Figure S6. TGA traces of the pristine fibers (blue), the dopamine coated fibers (green) and the N^+ coated fibers (purple).

7 Brunauer–Emmett–Teller (BET) measurements

We conducted BET measurements to determine the specific surface area of the pristine fibers, the polydopamine coated fibers and the polymer grafted fibers. The results are featured in **Table S2**.

Table S2. Surface area of the pristine and coated fibers determined via BET measurements.

Fiber	Surface area [m^2 g^{-1}]
Pristine	1.7579
Polydopamine	7.0914
Polymer grafted	5.3781

8 Adsorption Experiments

We conducted multiple adsorption experiments to determine the adsorption behavior of the coated and pristine stonewool fibers. First the coated and pristine fiber cubes were cut into pieces of approximately 30 mg (the accurate mass was determined for each cube individually) and placed in an LCMS vial. Next, 1.5 mL of an aqueous solution of PFOA with a known concentration was added and the samples were placed on a rotary wheel, which operated at a speed of 25 rpm. Samples were taken at 0, 1, 2, 5, 10, 30, 60, 180, 1,440, 3,120 and 4,320 minutes for the first experimental series, at a concentration of 2.5 g L^{-1} PFOA in water, and at 0, 1, 2, 5, 10 and 180 minutes for the concentration dependency study, which investigated the adsorption efficiencies of 0.5, 2.5 and 5 g L^{-1} PFOA solution. All samples were taken by drawing the solutions with a syringe and filtered using a syringe filter (PTFE, 0.22 µm) before preparing the samples for the NMR investigations, which were used to determine the amount of PFOA removed and are discussed in the next section.

9 Nuclear Magnetic Resonance (NMR) Spectroscopy

^{19}F NMR spectroscopy was used to determine the concentration of PFOA in each of the adsorption experiments. To the analyte solution (450 µL), 50 µL of a 48 mg mL^{-1} solution of $Cr(acac)_3$ in water was added as a relaxation agent. Next, 50 µL 6 mM solution of 3,5-difluorobenzoic acid was added as an internal standard to allow the quantification of the PFOA concentration. Finally, 50 µL D_2O was added as a lock solvent. In the presence of $Cr(acac)_3$, the longest T_1 of the ^{19}F nuclei in the samples was 188 ms as determined by an inversion recovery experiment.

Accordingly, the NMR experiment utilized a 90 pulse, 1 s acquisition and 0.5 s relaxation delay. Samples were measured at 25°C. All samples were processed in MestreNova using baseline correction (Whittaker smoother), phase adjustment, and exponential apodization (10 Hz, based on the native peaks widths of >20Hz). Exemplary ^{19}F NMR spectra of the 5 g L^{-1} PFOA at $t = 0$, and after removal with both the pristine and coated fibers are shown in **Figure S7**.

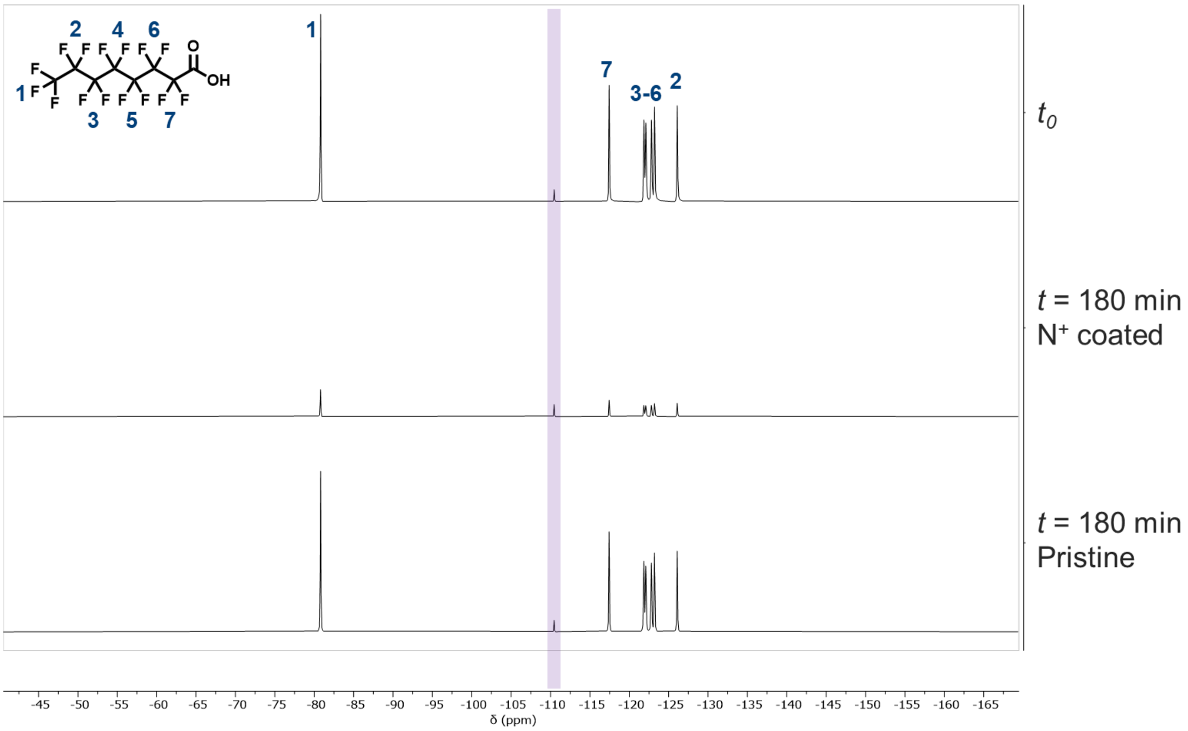

Figure S7. ^{19}F NMR spectra of PFOA at t_0 of a 5 g L^{-1} concentration and after removal for 180 min with the N^+ coated fibers and the pristine fibers (H_2O/D_2O, 376 MHz). The highlighted resonance is associated with the 3,5-difluorobenzoic acid internal standard.

First, a calibration curve was acquired using 0.2, 0.5, 1, 2, 2.5 and 5 g L^{-1} concentrated PFOA samples, depicted in **Figure S8**.

The average integral of each fluorine resonance per fluorine relative to the internal standard was plotted against the known concentration of the solution (black squares) and fitted with a linear function (red line). The concentration of the unknown samples were subsequently determined using the slope, $\boldsymbol{b}$, and the intercept, $\boldsymbol{a}$, in the equation:

$$\log_{10} C_{\mathrm{PFOA}} = \frac{\log_{10}(\mathrm{Integral(F)}) - a}{b}$$

Where C_{PFOA} is the concentration of PFOA and Integral(F) is the average integral of each fluorine resonance per fluorine.

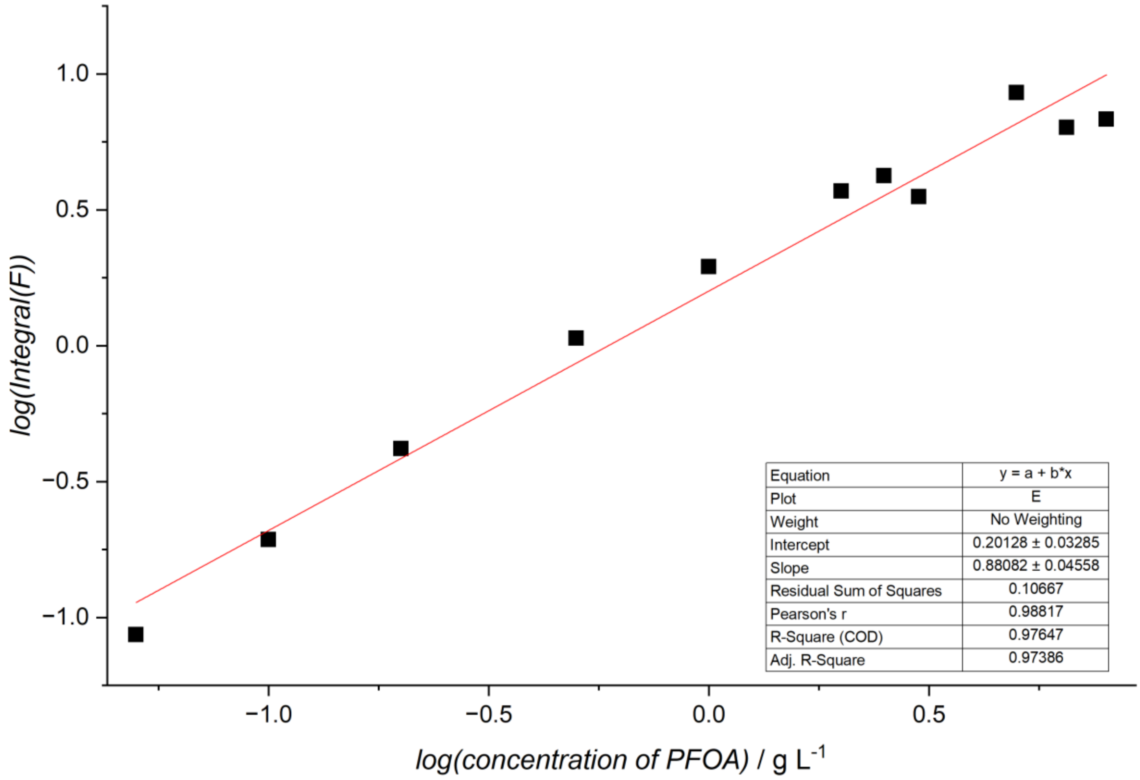

Figure S8. Calibration curve of known PFOA concentrations (0.05, 0.1, 0.2, 0.5, 1, 2, 2.5, 3, 5, 6.5, and 8 g L^{-1}).

Subsequently, the adsorption efficiency was investigated via the adsorption experiments described in section 6. Initially, we investigated the kinetics of the removal of a 2.5 g L^{-1} concentrated PFOA solution; the results are featured in **Figure S9**. As we observed a plateau at the 3 hour datapoint for both the coated and the pristine fibers, we decided to reduce the number of datapoints to 1, 2, 5, 10 and 180 minutes for a concentration study featuring the removal of a 0.5, 2.5 and 5 g L^{-1} concentrated PFOA solution.

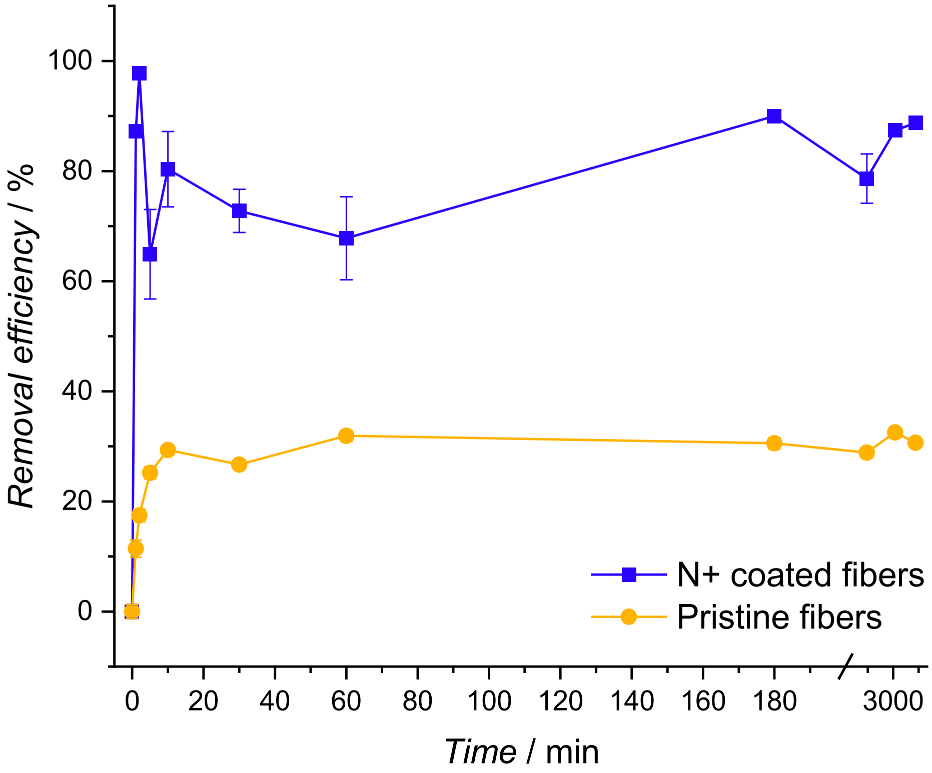

Figure S9. Time dependent removal of PFOA (2.5 g L^{-1}) by polymer grafted (blue) and pristine fibers (yellow). The error bars show the variations in integrals of each of the ^{19}F NMR references.

10 Adsorption Isotherm

To gain deeper knowledge into the adsorption process of the uncoated and polymer grafted stonewool fibers we conducted a study where we varied the concentrations of PFOA (a 0.05, 0.1, 2.5, 5, 6.5 and 8 g L^{-1}) and investigated the amount adsorbed after 10 minutes to be able to establish the Langmuir sorption isotherm.

Firstly, we calculated the adsorption capacity (q_e) with the equation shown below.

$$q_e = \frac{(C_0 - C_e) \cdot V}{m}$$

where C_0 is the initial concentration in mg L^{-1}, C_e is the equilibrium concentration of the adsorbate in solution in mg L^{-1}, V is the volume of the solution in L, and m is the mass of the absorbent in g. The plotted data for the adsorption capacities are shown in **Figure S10**.

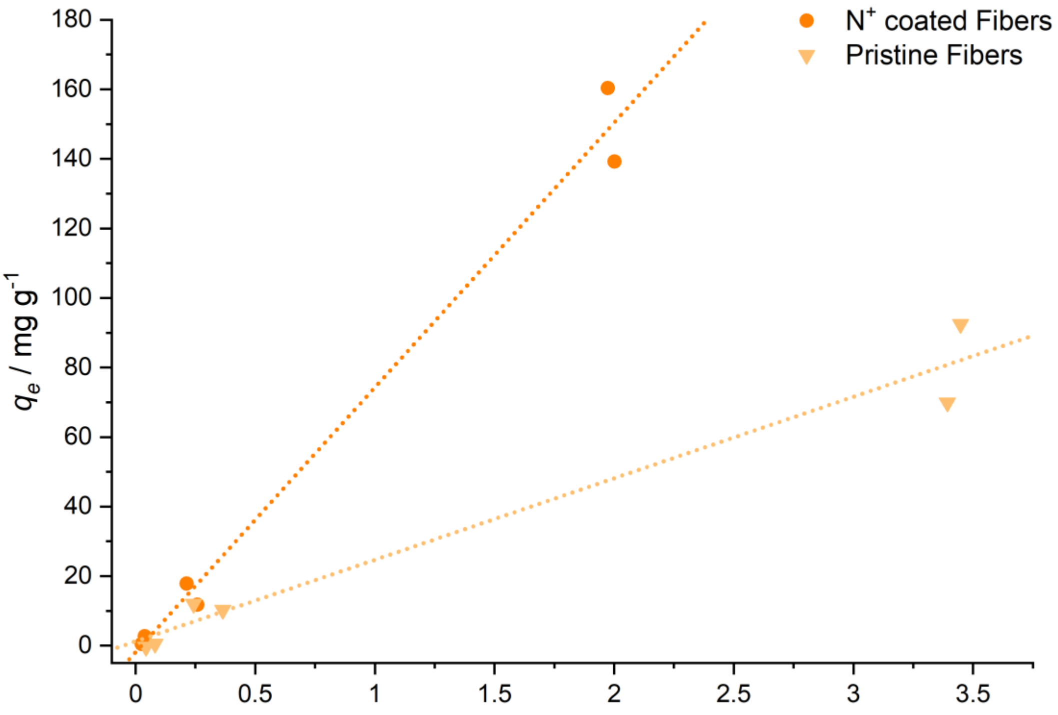

N$^+$ coated Fibers

Equation	y = a + b*x
Plot	qe [mg/g]
Weight	No Weighting
Intercept	-1.86957 ± 4.79973
Slope	76.14544 ± 4.15178
Residual Sum of Squares	318.97498
Pearson's r	0.99411
R-Square (COD)	0.98825
Adj. R-Square	0.98531

Pristine Fibers

Equation	y = a + b*x
Plot	P qe [mg/g]
Weight	No Weighting
Intercept	1.22548 ± 4.30598
Slope	23.45309 ± 2.17125
Residual Sum of Squares	264.80923
Pearson's r	0.98329
R-Square (COD)	0.96685
Adj. R-Square	0.95857

Figure S10. Adsorption isotherms of the pristine (yellow) and the polymer grafted fibers (orange) including the fit to the Langmuir model (dashed lines).

11 References

[1] A. C. Morrissey, V. Jayalatharachchi, L. Michalek, P. Egodawatta, N. Zaquen, L. Delafresnaye, C. Barner-Kowollik, *RSC Appl Polym* **2024**, *2*, 490-496.

[2] C. M. Preuss, T. Tischer, C. Rodriguez-Emmenegger, M. M. Zieger, M. Bruns, A. S. Goldmann, C. Barner-Kowollik, *J Mater Chem B* **2014**, *2*, 36-40.
